# The Effect of Losses Disguised as Wins and Near Misses in Electronic Gaming Machines: A Systematic Review

**DOI:** 10.1007/s10899-017-9688-0

**Published:** 2017-04-18

**Authors:** K. R. Barton, Y. Yazdani, N. Ayer, S. Kalvapalle, S. Brown, J. Stapleton, D. G. Brown, K. A. Harrigan

**Affiliations:** 1 Department of Psychology, University of Waterloo, Waterloo, Canada; 2 Department of Kinesiology, University of Waterloo, Waterloo, Canada; 3 Department of Recreation and Leisure Studies, University of Waterloo, Waterloo, Canada; 4 Gambling Research Lab, University of Waterloo, Waterloo, Canada; 5 University of Waterloo Library, Waterloo, Canada; 6 David R. Cheriton School of Computer Science, University of Waterloo, Waterloo, Canada

**Keywords:** Losses disguised as wins, Near misses, Systematic review, Gambling, Behaviour, Psychophysiology

## Abstract

Near misses and losses disguised as wins have been of interest to gambling researchers and policymakers for many years (e.g., Griffiths in J Gambl Stud 9(2):101–120, 1993). This systematic literature review describes the behavioural, psychological, and psychobiological effects of near misses and losses disguised as wins (LDWs) in an effort to evaluate their precise influence on the player and to highlight areas requiring further investigation. A systematic search for relevant studies was conducted using Scopus, PubMed, PsycINFO, ProQuest Sociology databases, and the Gambling Research Exchange Ontario Knowledge Repository. A total of 51 (from an initial pool of 802) experimental peer-reviewed studies using human participants were found between 1991 and 2015. The systematic review revealed that near misses motivate continued play, but have varying effects on the emotional state or betting behaviour of the player. Near miss events were also shown to be associated with elevated skin conductance levels and diffuse activity across the brain, most consistently in areas processing reinforcement and reward. Re-examination of the studies of near misses events after classifying the type of game feedback suggested that the effectiveness of near misses is related to the phenomenology of a near miss itself rather than as a response to auditory or visual feedback provided by a slot machine. In contrast to near misses, the presence of LDWs was found to relate to an overestimation of how much a player is actually winning and was consistently viewed as an exciting event. The effect of LDWs appears to be driven by the presence of visuals and sounds most often associated with a true win. Practical implications and directions for future research are also discussed.

**Electronic supplementary material:**

The online version of this article (doi:10.1007/s10899-017-9688-0) contains supplementary material, which is available to authorized users.

## Introduction

Slot machines and other forms of electronic gaming machines (EGMs) are widely accessible all over the world; they can be found in casinos, racetracks, bars, and even airports. The ease of access to EGMs is of considerable concern as EGMs have been associated with higher rates of problem gambling than other more accessible methods of gambling, such as bingo and scratch cards (Breen and Zimmerman 2002). The presence of EGMs has also been associated with a greater incidence of gambling as a whole in the general population (Urbanoski and Rush 2006). These and other findings have caused researchers to speculate that EGMs are the most addictive (Dowling et al. 2005) and problematic (Götestam and Johansson 2003) form of gambling available to the public, with some players equating EGMs to “crack cocaine” (Breen and Zimmerman 2002).

### The Popularity of Electronic Gaming Machines

Worldwide, slot machines and EGMs represent a large and highly profitable segment of the gambling industry. Internationally, across major gambling locales, such as Macau, Atlantic City, and New Jersey, a relatively small number of EGMs are responsible for a disproportionately high amount of revenue for casinos. In Atlantic City in 2015, across all casinos, 1.73 billion USD, approximately 71% of the revenue for all casinos in the city, was generated by an average of 16,384 slot machines (New Jersey Casino Control Commission 2015). In Nevada casinos, including the Las Vegas Strip, 167 k slot machines generated 7.08 billion USD, approximately 63% of all casino revenues (Nevada Gaming Control Board 2016). In Macau in 2016, approximately 1.42 billion USD in revenue was generated from the operation of 13,826 EGMs (Gaming Inspection and Coordination Bureau Macao 2016).

In addition to being a highly profitable segment of the international gambling industry, EGMs have been identified as the preferred type of casino game by casino visitors in the United States, with 61% of visitors naming EGMs as their favourite (American Gaming Association 2013). EGMs also appear to attract a higher proportion of players afflicted with depression than other forms of gambling (Blaszczynski and Nower 2002). A series of observational studies have also shown that the average difference between when people participants started gambling versus when the participants first reported for treatment for problem gambling was significantly shorter than other forms of gambling (Breen and Zimmerman 2002; Breen 2004).

A key reason for the addictiveness of EGMs may be found in the “rapid, continuous, and repetitive nature” of the games (Breen and Zimmerman 2002). Players appear to be drawn to specific, conspicuous characteristics in EGMs that are intentionally included in the games to make them more enjoyable and engaging—ranging from subtle cues meant to induce frustration or excitement, to the more obvious visual and auditory feedback provided throughout gameplay. These sorts of structural characteristics of EGMs have been argued to lead to the acquisition, development, and perpetuation of the desire to engage in a form of gambling (Griffiths 1993). For example, when a player wins, the arousal inherent to winning may be compounded by the lights and sounds produced by the EGM, establishing classical conditioning and causing players to chase wins so they can experience this arousal again. In addition, EGMs often have more subtle mechanisms introduced that are designed to encourage a player to keep playing. By examining probability accounting reports (PAR sheets, the manufacturing design documents describing the mathematical and algorithmic underpinnings of an EGM game), Harrigan and Dixon (2009) found that the outcome of each spin on an EGM may be biased to provide a more exciting experience, such as the experience of “almost winning” or nearly missing a win at a rate much higher than expected by chance, providing a more engaging experience than an outright loss.

To date, while a number of specific structural characteristics have been identified in EGMs that encourage play, our understanding of *how* they influence the gamblers and reinforce play is poor. In this systematic review we focus on two salient features present in many slot machines and EGMs: (1) near misses and (2) losses disguised as wins (LDWs). To better understand the influence of these two features on gambling behaviour, we focused on their behavioural, psychological, and psychobiological effects on both healthy and problem gamblers. We provide a clear and up-to-date understanding of the effects of near misses and LDWs, sufficient not only to guide future research in the field, but also to influence regulatory policy.

### Near Misses

**Fig. 1:**
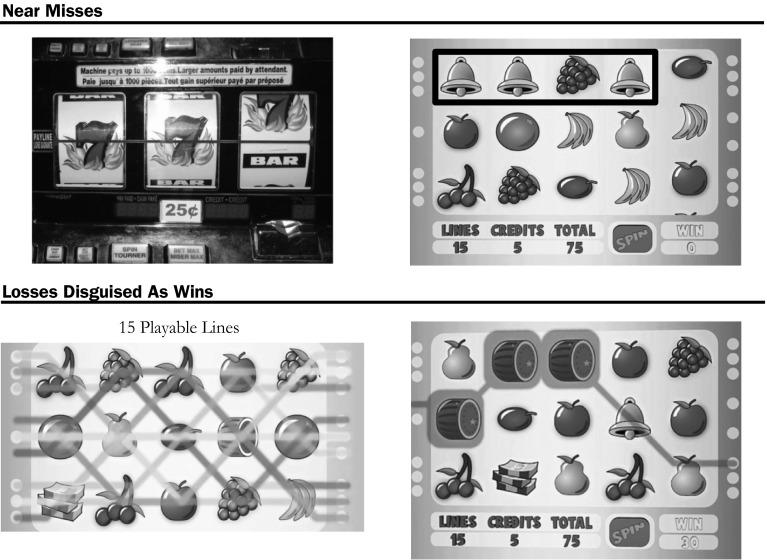
Examples of near misses and losses disguised as wins (LDW). In the *top row*, two forms of near misses are presented: (*left*) a 3-reel slot machine with a near miss above the payline; (*right*) a near miss on a 5-reel slot machine (highlighted in *black*). In the *bottom row*, a multiline EGM with 15 playable lines (*left*) is presented and an example of an LDW (*right*). In this example, by betting 75 credits, on 15 lines at 5 credits each, the player’s win of 30 is 45 credits lower than the cost of play, presenting as a LDW

Examples of two traditional types of near misses on an EGM are provided in Fig. 1. In the first type of near miss, often encountered on 3-reel EGMs, two jackpot symbols appear on the payline, and a third stops just above or below the payline. In the second type, depicted on a 5-reel EGM, two jackpot ‘bell’ symbols are adjacent to each other on a played line, but separated from a third ‘bell’ by a ‘grapes’ symbol, preventing a win. To the gambler, this may feel like he or she was close to winning (Horton et al. 2006). Near misses are often the product of virtual reels (a mechanism sometimes termed as weighted reels), which bias the outcome of the game toward showing symbols adjacent to high paying jackpot symbols on certain reels. Skinner provides one potential explanation for the effectiveness of near misses in *Science and Human Behavior* (1953), “Almost hitting the jackpot increases the probability that the individual will play the machine, although this reinforcement costs the owner of the device nothing” (Skinner 1953, p. 397).

### Losses Disguised as Wins

Losses disguised as wins (LDWs) occur when a player wins less money than they bet, resulting in an overall loss. Modern multiline slot machines celebrate LDWs in a similar or identical way to that of a true win. An example LDW is in the bottom row of Fig. 1: the player bets 75 credits and receives 30 back. LDWs typically occur in multiline slot machines, where the player is playing a small amount on each line, and small wins occur on some of the lines, but not enough to make an overall win. The EGM’s use of winning sounds and winning animations appear to have a strong influence on a player’s urge to continue playing, masking the overall loss (Dixon et al. 2010).

### Implications of the Systematic Review

Our systematic review added several important elements to our understanding of the impact of structural characteristics of EGMs. Near misses were found to be significantly more arousing, motivating, and frustrating than losses. Of 11 studies, 10 showed that near misses were associated with large skin conductance responses, a traditional indicator of physiological arousal (Lang et al. 1993). Analysis of brain activity showed that near misses produce activity in areas related to reward and uncertainty, reinforcing the mechanism through which they may act on the player. Problem gamblers were found to show elevated brain activity in response to a near miss in parts of the brain associated with emotional regulation (Goldin et al. 2008), while casual gamblers did not show this effect.

Our review of research on LDWs provides key insights into the type and character of the cognitive distortion induced by LDWs. Generally, the frequency of LDWs was associated with an overestimation of how much one is winning and appears to be brought about by the celebratory sounds and visuals accompanying the LDW. The visual and auditory stimuli were also found to contribute to elevated arousal, as indexed by skin conductance level. Problem gamblers were found to prefer games offering LDWs more than non-problem gamblers.

We also found some evidence, both neurological and physiological, of the tendency for problem gamblers to become less aroused by near misses or LDWs than non-problem gamblers.

Throughout our review, we highlight inconsistent findings and questions of theoretical interest raised by our systematic review. Additionally, by providing an up-to-date understanding of these game characteristics, we hope to provide regulators with sufficient evidence to understand the role of near misses and LDWs in producing gambling behaviour. This is particularly noteworthy in light of the Australian government (Queensland Government 2015) banning the intentional design of near misses in games, a move that appears justified based on the results presented here.

## Methods

The following framework was employed to conduct the systematic review: (1) formulated the research questions; (2) defined the inclusion and exclusion criteria for study selection; (3) conducted the systematic literature search; (4) screened search results through title, abstract and full text; (5) extracted relevant evidence from the included studies; (6) synthesized and summarized evidence. Our review team consisted of knowledge experts in the fields of gambling research, systematic reviews, psychology, consumer behaviour, as well as two information specialists.

The systematic review investigated two research questions: what are the effects of the near misses/LDWs on the player? and Does the gambling status of a player (i.e., normal, at-risk, or problem gambler) alter the effect of near misses/LDWs on the player? Effects were defined as changes in psychological or cognitive state, behaviour, or psychobiology. Finally, data were examined for the effects of specific aspects of how the EGMs provide feedback to identify key characteristics that may be driving the response to near misses and LDWs.

### Literature Search

A systematic literature search for relevant studies was conducted in November, 2015 using Scopus, PubMed, PsycINFO(PsycNet), and Proquest Sociology databases as well as the Gambling Research Exchange Ontario Knowledge Repository—Synopsis collection which contains concise summaries of peer-reviewed gambling research articles. Comprehensive search methods were developed by the information specialists in consultation with the research team. Search strategies consisted of author keywords (those appearing in the title or abstract of the paper) and subject headings (controlled vocabulary specific to each database) focusing on two sets of search terms: (1) the structural characteristics of EGMs of interest: near miss, just missed, almost winning, just missing, narrow win, virtual reel, losses disguised as wins, multiline, multiple paylines, and small wins; and (2) gambling-related terms intended to focus the review on the effects of EGMs alone, with culturally-appropriate terms for EGMs from a variety of nations: gamble, lottery, gaming, pokies, poker, slot, and fruit machines. The primary and secondary search terms were combined using the logical AND, OR, truncation and wildcard, and WITHIN operators to identify all pertinent studies within each research database.

Full search strategies are available as electronic supplementary material.

### Study Selection

Studies selected for inclusion in the review were required to be published, peer-reviewed, written in English, include experiments on human participants, and using an EGM or simulation of an EGM. Studies were included if they specifically addressed the effects of near misses or LDWs in gambling on single line slot machines, multiline slot machines, or computer generated simulations of either single or multiline slot machines. In addition, studies were required to quantify the effect on the player either psychologically (i.e., self-report), behaviourally (e.g., reaction times, amount gambled, etc.), or psychophysiologically (e.g., skin conductance, heart rate, etc.). Studies that were either randomized controlled trials or observational in nature were included in the review. The relevance of each study identified by the literature search was assessed in two stages, by title and abstract screening and by full text screening. A screening tool was developed by the research team to guide reviewers in both stages of the relevance assessment process. To ensure reliability of the screening tool, the screening tool was piloted separately on ten randomly selected publications from the literature search for both the title and abstract screening and full text screening steps. Each title and abstract, and later full text, were reviewed by two independent readers using the screening tool. At both stages of screening, the decision to include or exclude a study was made by reaching consensus. In cases where consensus could not be reached, the appropriate knowledge experts on the research team were consulted to make the final decision.

### Data Extraction and Review

Data and evidence were extracted from all included studies through the use of a data extraction tool. The data extraction tool was developed by the research team to guide each reviewer throughout the data extraction stage. The data extraction tool used the following categories: primary author, jurisdiction, study design, sample size, population employed, type of gambling task studies, topic of research (either near miss or LDW), a description of psychological, physiological, or behavioural outcome or effect, concluding remarks, and recommendations for policy makers and practitioners. To ensure reliability of the data extraction tool, the tool was piloted on ten randomly selected studies.

The evidence collected by the data extraction was summarized using thematic analysis, identifying all consistent and unique effects of near misses and LDWs on the player. Due to the sizable diversity in the methods, types of collected data, and populations studied within each included study, a meta-analysis of the results was not possible.

## Results

### Results of Screening

**Fig. 2:**
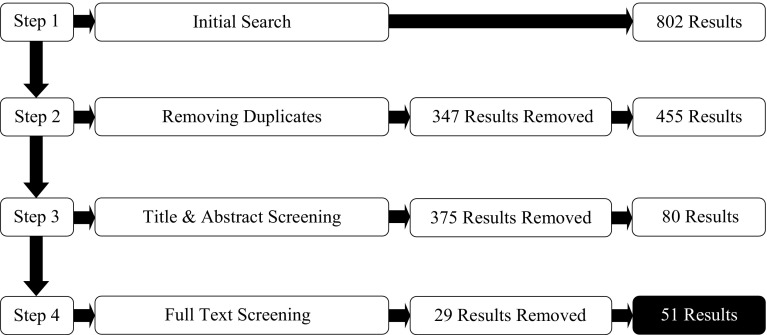
Flow diagram showing inclusion and exclusion of the studies identified through the database search

A schematic of the screening process is presented in Fig. 2. The literature search identified an initial pool of 802 studies. The initial pool of studies was reduced by removing duplicates items, resulting in 455 studies being retained for screening. The studies were first subjected to a title and abstract screening, assessing the degree to which each study met inclusion criteria from the title and abstract matter alone, followed by a screening of the full text for all studies that passed title and abstract screening. Of the 455 studies screened, 375 did not match the inclusion criteria following text and abstract screening and a further 29 studies were excluded after the full text review. After completion of the screening process, 51 studies (40: Near Misses; 10: LDWs; 1: Both) were included in the final review.

Characteristics of the included studies are summarized in Table 1. Studies addressing near misses were conducted worldwide, with the majority of these studies being performed in the United Kingdom (34%) and United States (27%), with a moderate number emerging from Australia (10%) and Canada (10%). Of these studies, the majority used some form of simulated EGMs (71%; consisting of two or more reels) to study the topic of near misses, with the minority of studies using commercial EGMs (20%) or VLTs (2%), and the remainder using other types of simulated gambling (7%; Wheel of Fortune task or simulated roulette task). Four of these studies were observational in nature, with the remaining 37 reported as some form of controlled experiment. Studies were published between 1991 and 2015, with 62% of the studies published since 2010. For studies examining LDWs, the majority of studies were conducted in Canada (81%), with one each in Australia and the United Kingdom. Five studies examining the effects of LDWs were conducted using commercially available EGMs, while six used simulated EGMs. The LDW studies were conducted between 2010 and 2015.

**Table 1:** The characteristics of the 51 studies included in the systematic review

	Focus			
	NMs	LDWs	Both	Total
Location				
Australia	4	1	–	5
Canada	4	9	–	13
China	2	–	–	2
Germany	1	–	–	1
Hungary	1	–	–	1
Spain	1	–	–	1
United Kingdom	13	–	1	14
United States	11	–	–	11
Multiple sites^a^	3	–	–	3
Type of study design				
Experiment (randomized)	18	6	1	25
Experiment (non-randomized)	4	–	–	4
Experiment	13	4	–	17
Multiple experiments	1	–	–	1
Observational study^b^	4	–	–	4
Gambling Task				
EGM	5	5	–	10
Rapid-roulette	1	–	–	1
Simplified two reel slots	1	–	–	1
Simulated EGM	29	5	1	35
Video lottery terminal	1	–	–	1
Wheel of fortune task	3	–	–	3

Only those studies that clearly outlined the experimental design or task design were categorized as such. Studies that did not clearly indicated if they were randomized were categorized as experiments. The gambling task was determined in accordance with the description provided in the original study

^a^Collaborative works by researchers from different jurisdictions

^b^Field and exploratory studies

### The Effects of Near Misses on the Player

The results reported by each study were extracted and classified as being either behavioural, psychological, or psychobiological. Any findings specific to the effect of near misses on problem or at-risk gamblers or problem gamblers (through either clinical diagnosis or scoring on a gambling severity scale) were examined separately. The influence of the way the gambling task was designed to respond during play, specifically that of the type of the game feedback, was also examined to potentially account for any reported differences in how near misses affect the player.

#### Psychological Effects

Four studies (Gibson and Sanbonmatsu 2004; Dixon and Schreiber 2004; Dixon et al. 2009; Dymond et al. 2014) described the effect of near misses on the perception of winning. Two of these studies (Gibson and Sanbonmatsu 2004; Dixon et al. 2009) reported that players tend to report significantly more wins when experiencing near misses. Gibson and Sanbonmatsu (2004) also showed that self-reported optimists were also found to report marginally more near misses than self-reported pessimists, but no significant difference was observed for true wins. Dixon and Schreiber (2004) found that players verbally responded to near miss events more similarly to wins than losses. In two separate samples of players, Dymond et al. (2014) reported that players rated near miss events as closer to a win event than that of a loss. The attribution of near misses as wins was shown to be responsive to conditional discrimination training in 10 out of 16 participants (Dixon et al. 2009).

Sixteen studies showed evidence for near miss events to influence a player’s thoughts or emotions. Of these studies, nine reported that the presence of near misses affected the motivation to play (Clark et al. 2009; Qi et al. 2011; Billieux et al. 2012; Clark et al. 2012; Porchet et al. 2013; Devos et al. 2015; Sharman et al. 2015) or persist in play (Clark et al. 2012, 2013). Billieux et al. (2012) reported that the persistence to continue playing following a near miss was associated with a reduced feeling of self-control on the outcome of the game. Devos et al. (2015) reported that the motivation to continue gambling following a win or near miss was related to the time it took the gambler to respond following either event, with lower reaction time predicting ratings of the desire to continue to play and persistence of play in hierarchical regression. Of the nine studies that assessed the type of emotional response to near miss events, six of studies reported that near misses were associated with either less of a feeling of reward than compared to a win (Lole et al. 2013) or were rated as less pleasant or more aversive than wins or losses (Griffiths 1991; Clark et al. 2009; Qi et al. 2011; Sharman et al. 2015; Sharman and Clark 2015). Two studies reported no difference between near miss events and losses, but did report that wins were rated as significantly more pleasant than losses (Clark et al. 2014; Ulrich and Hewig 2014) and one study found that near misses occurring before the payline were found to be rated as more pleasant than other types of losses (Clark et al. 2012). In terms of observable emotional response, as measured by facial electromyography, nearly winning a big jackpot in the Wheel of Fortune task (Wu et al. 2015) and nearly missing a jackpot in a simulated EGM task (Sharman and Clark 2015) were associated with an increase in zygomaticus activity (muscles of expression in the cheek that allow clenching of the jaw or the expression of happiness), similar to the type of response following a win event. An increase in corrugator activity (muscles allowing expression using the eyebrows, such as sadness, anger, and fear) was reported to occur as a response to win events by Sharman and Clark (2015) and to loss events by Wu et al. (2015).

Three studies investigated whether near miss events influence higher level cognitive processes or constructs. Luo et al. (2011) reported that players reported higher levels of impulsivity and willingness to engage in risky behaviour following a near miss during slot machine gambling. Two studies investigated the relationship between near miss events and perceived luckiness using the Wheel of Fortune task (Wohl and Enzle 2003; Wu et al. 2015). Spins that nearly missed a big win were associated with lower ratings of luckiness in one study (Wu et al. 2015) and increased ratings of luckiness in another study (Wohl and Enzle 2003). However, narrowly missing a big loss was associated with an increased rating of luckiness in both studies.

#### Behavioural Changes

Three studies investigated whether the presence of near miss events influenced which EGMs players selected to play on or which symbols they decided to hold. Clark et al. (2012) reported that the presence of near miss events did not contribute to the expectation to win or influence which of the reels the participants chose to hold during EGM play. Two studies found that players were unable to differentiate between several EGMs offering different rates of near misses (Maclin et al. 2007; Kurucz and Körmendi 2012). Maclin et al. (2007) also reported a tendency for players to prefer EGMs offering near misses when they experienced strings of losses.

Three studies evaluated the influence of near miss events on the post-reinforcement pause (PRP) time following the outcome of the game. One study by Belisle and Dixon (2015) reported that players showed a tendency to pause for longer following a near miss relative to a loss, with the effect made stronger by the number of matching symbols visible on the screen at the time of the event. In contrast, Dixon et al. (2013) observed that the length of pause following a near miss was significantly shorter than those during a win or loss. No difference in the pause time was found following a near miss event from that of a win or loss in one study (Worhunsky et al. 2014).

Eight studies assessed the influence of the presence of near misses on how people gambled across a session. No effect on the number of times a player gambled was observed in three studies (Whitton and Weatherly 2009; Sundali et al. 2012; Devos et al. 2015). Similarly, no consistent effect was found on betting behaviour, with one study reporting no difference in bet on the next spin following a near miss (Wu et al. 2015), one study reporting the tendency to bet less on the next spin (Sundali et al. 2012), and one study finding that players bet more following near miss or win than when experiencing a loss (Alicart et al. 2015). Additionally, the presence of near misses was associated with a tendency to extend the gaming session in three studies (Griffiths 1991; Kassinove and Schare 2001; Côté et al. 2003).

#### Psychobiological Effects

In total, eleven studies examined the effect of near misses on physiological arousal of players by measuring skin conductance level (SCL, sometimes referred to as electrodermal activity, differentiable into tonic and phasic components; *N* *=* *10*) and heart rate (*N* *=* *6*). Of the ten studies that assessed the responsiveness of SCL to near miss events, the majority of studies demonstrated the tendency for SCL to elevate in response to a near miss (Griffiths 1990; Dixon et al. 2011, 2013; Clark et al. 2012, 2013; Porchet et al. 2013; Sharman and Clark 2015). One study showed a significant correlation between self-reported levels of excitement following a near miss and SCL (Lole et al. 2012). One study showed that only wins produced elevated SCL (Wilkes et al. 2010). In contrast, out of the six studies that assessed heart rate variability (HRV), two studies found evidence for a deceleration of heart rate following near misses (Dixon et al. 2011; Porchet et al. 2013), two studies found no effect of near misses (Wilkes et al. 2010; Lole et al. 2012) and two study found evidence for acceleration of heart rate following near miss events (Griffiths 1991; Clark et al. 2012).

Several studies assessed the real-time response of the brain to near misses using electroencephalography (EEG). Lole et al. (2015) reported that parietal clusters of electrodes were associated with near misses and increased feedback-related negativity (FRN), whereas wins were associated with increased frontal electrode activity and feedback-related positivity (FRP). FRN and FRPs are specific negative or positive deflections in the EEG waveform in response to visual or auditory feedback (see Miltner et al. 1997 for more details). Significantly elevated FRN was also reported by Ulrich and Hewig (2014) in addition to significantly higher P300 amplitude following near misses. Elevated P300 was reported for wins and near misses by Alicart et al. (2015) but not for losses. EEG band-power in the alpha, low beta, theta, and beta-gamma ranges were also found to be elevated for near misses and wins but not losses (Alicart et al. 2015). However, inconsistent with these findings, Lole et al. (2013) reported the tendency to produce a reduced FRN response following near miss rather than the elevation reported in the majority of the EEG studies.

In terms of the brain areas subtending the response to near miss events, five studies assessed brain activity using functional magnetic resonance imagery (fMRI). Diffuse activity, including significantly higher blood oxygenation level dependent (BOLD) signal in the prefrontal cortex, insular cortex, inferior frontal gyrus, medial frontal gyrus, and bilateral inferior thalamic activity was observed by Dymond et al. (2014) in response to near misses. Habib and Dixon (2010) reported unique activity in the inferior parietal lobule to near misses relative to losses. Shao et al. (2013) reported diminished positive BOLD signals in the ventral striatum and amygdala in response to near misses relative to wins. Worhunsky et al. (2014) reported increased BOLD activity in occipital, posterior cingulate, and inferior and superior parietal cortex in response to near misses. Clark et al. (2009) reported increased striatum and insular cortex activity in response to near misses. One study attempted to directly relate the role of the insular cortex in motivating continued play when experiencing near misses by comparing patients with insular cortex lesions to healthy controls (Clark et al. 2014). They reported that lesions to the insular cortex but not the amygdala were necessary to abolish the motivation response to near misses.

#### Effects on Problem or At-Risk Players

The potential effect of near misses on problem or at-risk gamblers, both psychologically and physiologically, was examined by nine studies. In one study of the physiological response following a near miss, problem gamblers were not shown to differ from healthy controls in their SCL responses or post reinforcement pause times (Dixon et al. 2013).

Six studies assessed the neural activity subtending the response to a near miss event in pathological or problem gamblers. Using EEG, Lole et al. (2015) reported evidence for a reduced FRN response to losses and reduced FRP to wins in pathological gamblers, but showed that the FRN response to near misses did not differ from that of healthy controls. Five studies used a neural imagery technique, such as fMRI or magneto-encephalography (MEG) to explore differences between healthy and pathological gamblers. Unique brain activity in response to a near miss was reported in the right occipital gyrus, right uncus extending into the amygdala, midbrain, and cerebellum, in one study (Habib and Dixon 2010). Chase and Clark (2010) reported that midbrain activity in response to near miss outcomes was predicted by the degree of gambling severity in a regression analysis. The authors also reported that problem gamblers showed an increased response in the striatum to both wins and near misses relative to healthy controls. Worhunsky et al. (2014) reported significantly elevated brain activity in pathological gamblers in the occipital, posterior cingulate, and inferior and superior parietal cortices in response to near misses when compared against healthy controls. Van Holst et al. (2014) showed that near misses increased activity in the ventral striatum, bilateral insular cortex, and was significantly correlated with degree of problem gambling. In terms of real-time activity, one study showed through the use of MEG that increased theta power in the insular and orbitofrontal cortices was correlated with the gambling severity (Dymond et al. 2014).

Only one study evaluated whether the behavioural response to near misses could be reduced in pathological gamblers through a clinical intervention, an acceptance and commitment intervention (Nastally and Dixon 2012). Using a series of informational slides providing information on near misses and separate mindfulness exercises, the authors reportedthat self-reported proximity to win at each near miss event could be reduced through an intervention of this type.

#### Differences Accounted for by Game Feedback

To determine whether any differences in the observed psychological, behavioural, or neurobiological responses to near misses across the sampled studies could be accounted for by the fidelity of the gambling task, rather than the phenomenon of a near miss itself, the way each study provided feedback about the outcome of the game was extracted from each of the sampled studies. Studies were classified as providing animated feedback, graphical feedback, auditory feedback, and/or text-based feedback to the participant. Of the sampled studies, 19 studies did not specify how the game outcome was presented to the player, 12 studies provided text-based feedback (such as, “You win!” or “You lose!”) on the screen, 7 studies provided feedback in the form of an animation or change in the visual display (such as flashing symbols on a jackpot), and 3 provided a physical payout on wins (cash or credits being handed to the player). In terms of auditory feedback, 9 of the 41 studies provided some form of auditory feedback throughout play of the game. Using this classification system, a thematic analysis identical to that of the overall analysis was used to identify whether game feedback produced any consistent psychological, behavioural, of neurobiological response. Despite sampling a reasonable range of feedback types, no consistent differences were observed to show that the type of game feedback altered player’s responses to the game above that of the perception of a near miss itself.

### Losses Disguised as Wins

As for near misses, the effects of LDW in each study were extracted and classified as being either behavioural, psychological, or psychophysiological. Studies independently investigating each of these effects in problem or at-risk gamblers were extracted and examined separately. Finally, to assess the influence of the specifics of the gambling task on producing any effect of LDWs, the effect of specific feedback features each the game were examined.

#### Psychological Effects

Five studies addressing LDWs identified the tendency for players to report inflated estimates of their win frequencies (Jensen et al. 2013; Dixon et al. 2014a, b; Dixon et al. 2015; Templeton et al. 2015). Of these, two of these studies found that the tendency to overestimate winning was influenced by the number of LDWs experienced throughout the play session (Jensen et al. 2013; Templeton et al. 2015).

Only one study presented evidence for the effect of LDWs on a player’s emotional state. Sharman et al. (2015) reported that in players experiencing both LDWs and near misses in the same game, the negative emotional valence associated near miss events was magnified by the presence of LDWs. Players experiencing LDWs without the presence of near misses were found to report significantly higher levels of enjoyment and motivation than those who did not experience LDWs.

#### Behavioural Changes

Three studies provided evidence of the tendency of multiline EGMs, allowing for LDWs, to influence which machines gamblers prefer to play on. Dixon et al. (2014a, b) reported that 94% of players preferred to gamble on multiline EGMs. Another study revealed that gamblers prefer multiline EGMs to single line EGMs, when afforded a choice between the two (Templeton et al. 2015). Players were also reported to be able to consciously plan how they play on multiline EGMs, maximizing the number of LDWs over losses by betting on more lines at once (MacLaren 2015). In addition to potentially influencing game selection, the presence of LDWs resulted in longer post-reinforcement pausing in two studies (Dixon et al. 2014a, b; Templeton et al. 2015).

#### Psychobiological Effects

No consistent effect of the presence of LDWs on heart rate and heart rate variability (HRV) was observed across the three studies that examined them. Slower heart rate was observed for larger LDWs than compared to smaller LDWs in one study (Dixon et al. 2015). A significantly higher heart rate variability was also observed in LDWs relative to real wins in another study (Dixon et al. 2014a, b). One study reported a significant elevation of HRV in response to wins but did not find this response in the case of LDWs (Dixon et al. 2010).

Three studies measured whether SCL was influenced by an LDW outcome. In one study, SCL was found to be elevated in response to a win or LDW but not a loss (Dixon et al. 2010). The size of the change in SCL was shown to be linearly related to the magnitude of the win or LDWs payout (Dixon et al. 2014a, b) and this did not significantly interact with the way that the wins, losses, or LDWs were presented to the player, in the form of auditory game feedback.

In contrast, one study did not find an effect of experiencing an LDW on the SCL response (Dixon et al. 2015).

#### Effects on Problem or At-Risk Players

Two studies investigated the role of gambling status in the effect of LDWs. In one study, high-risk gamblers, as identified by the PGSI (Problem Gambling Severity Index), were reported to prefer multiline EGM play more than single line EGM play relative to healthy controls (Dixon et al. 2014a, b). In a separate study, problem gamblers were shown to be hyposensitive to stimuli, showing reduced SCL in response to reward when gambling (Lole et al. 2014). Problem gamblers did not differ from healthy controls in the SCL response to losses or LDWs in this study.

#### Differences Accounted for by Game Feedback

The sound effects experienced when gambling on multiline EGMs shown to be associated with the tendency to overestimate wins in two studies (Dixon et al. 2014a, b, 2015). Both reinforcing visuals (Dixon et al. 2010) and sound (Dixon et al. 2014a, b) were significantly affected the player’s SCL when experiencing an LDW when physiological outcomes were measured in two studies.

## Discussion

This systematic review examined the psychological, behavioural, and psychobiological responses of individual players in response to near misses and LDWs across 51 studies published from 1991 to 2015.

For near misses, a number of consistent findings were observed. A number of studies suggest that near misses increase the frequency with which a player will estimate that they are winning and motivate continued play (nine studies); encourage longer play(three studies); lead to overestimation of the frequency of winning (four studies). Near misses also appear to result in an increase in SCL in a large number of studies (10 out of 11 studies). Near misses were also found to be viewed as negative or aversive events in 6 out of 9 studies. The present review also found no evidence for these effects to be the product of game feedback, suggesting that the response to near misses is a product of the phenomenology of seeing matching symbols alone and not some byproduct of how the EGM produces an exciting or engaging gameplay experience to generalize near misses as wins, such as through the use of visuals, animations, and sounds.

Notably, however, findings were not completely uniform across all the sampled studies, with considerable spread in the reported effects of near misses in a variety of different measured outcomes. For example, near misses were found to be associated with increasing one’s bet, decreasing one’s bet, or having no effect, each in a different study, making it difficult to determine whether near misses are capable of influencing per-play betting behaviour. The precise reasons for this inconsistency is presently unclear. However, existing work on gambling behaviour has found that the choice of behaviour is likely influenced by a number of individual (such as traits, motives, and gambling status) and situational (options to play, amount of money available, etc.) factors (Smith et al. 2007), many of which can vary greatly from player-to-player. Another inconsistent result was observed in how players respond emotionally to a near miss event, both in terms of self-reported measures and in the neuroimaging data. One likely possibility for the inconsistency in these studies is that the response to a loss event, or an event which is similar to a loss, like a near miss, is driven by more complex cognitive constructs (such as the degree of counterfactual thinking, see: Henderson and Norris 2013), leading to more varied responses when this and other variables are not accounted for. Other work has also shown that individual differences with response to gambling losses is strongly associated with the expectation of success and the degree to which the game is enjoyable or reinforcing (Campbell-Meiklejohn et al. 2008). Taken as a whole, betting and gambling behaviour appears to be the result of a potentially large number of different factors which vary between players, each of which must be better identified and measured in further studies so that the precise reason for the effect of near misses can more completely and accurately be understood. This is considered particularly important, in light of the consistent finding that EGMs featuring near misses appear to encourage or extend play—the underlying reason for the motivation to play remains unclear, at least at present.

Another area that requires further investigation is the ability for near miss events to produce a response in the player at the physiological level, such as is the case in heart rate and HRV outcomes. Despite finding that 10 out of 11 studies showed a significant elevation in SCL in response to a near miss, no such consensus was observed for heart rate or HRV and near misses. One reason for this may be that skin conductance responses are physical responses brought on by fundamentally different types of processing, but both occurring, at least in part, through activity in the autonomic nervous system. For example, when recording SCL during rest versus the performance of eight different tasks, tasks which captured both internalized processing (such as, solving complex arithmetic problems) and the processing of external stimuli (i.e., distinguishing between different levels of white noise), SCL was found to show a response in all tasks (Lacey et al. 1963). In contrast, heart rate was shown to decelerate in response to tasks requiring attention to external stimuli and accelerate in response to more internal processing. Other work has also suggested that heart rate may be less sensitive to certain kinds of emotional processing, such as that of sadness, than skin conductance measures (Kreibig et al. 2007). A study using simultaneously EEG, heart rate, and SCL measures in tasks requiring vigilance or sustained attention has shown that heart rate may be more sensitive to changes in overall vigilance in performing a task, whereas skin conductance was found to be associated with effort or time-on-task (Olbrich et al. 2011). Thus, heart rate and heart rate variability, rather than SCL, appear more strongly influenced by the type of processing being engaged in by the player in an average gaming session, something that can vary considerably across individual players. Future studies including these measures would profit from a more rigorous account of the types of processing (e.g., internal versus external processing) and degree to which the participant is attending to the gambling task, in addition to the existing practice of measuring self-reported emotional state and overall gambling severity level. Without more stringent control and investigation of gambling phenomenology, it is difficult to conclude how exactly near miss events are affecting the player, at a cognitive or neural level. This also suggests that conclusions raised from HR data, without further specification, should be interpreted with caution.

Diffuse activity in the brain reported across five studies of the effects of near misses, but a number of common areas were found to be active when processing near miss events, including the insular cortex (three studies), ventral striatum (two studies), and inferior parietal tissue (two studies), though activity was also reported in other areas in the brain, ranging from prefrontal tissue to occipital tissue. While each reported area is likely meaningful, in some way, to the context of gambling and the response to near misses, the less consistent activity may be the product of idiosyncrasies in task design or analysis regime. In terms of the most consistently significant activity in the insular cortex, inferior parietal, and striatum, these areas have often been implicated in the processing of uncertainty and in the assessment of reward or punishment status. For example, in a set of non-gambling tasks, the activity in the ventral striatum has been correlated with the magnitude of reward or punishment (Hsu et al. 2005) and is immediately separable from activity related to uncertainty or risk assessment. In contrast, activity inferior parietal, but particularly the inferior parietal lobule, appears related to the processing of uncertainty (Vickery and Jiang 2009). It is reasonable to conclude that near misses would recruit either of these modes of processing, so the observed activity is consistent with these other fields of research. The extent to which either uncertainty processing or reward/punishment assessment is engaged in when experiencing near miss events is a topic for further work. But the strongest and most convincing evidence for the direct role of this type of tissue or processing can be found in studies using patients with lesions, such as in Clark et al. (2014). In patients with lesions to the insula, the presence of a lesion significantly reduced the response to near miss events and their reinforcement on behaviour. Despite the relatively few studies identified by our systematic review addressing near miss events and the brain, it is clear that future work should further investigate the specific role of the insula and the complex interplay between tissue in the insular cortex, striatal, and inferior parietal lobule in processing near miss events.

In terms of real-time processing in the brain, consistent EEG signals (i.e., FRN, P300, and P3b) were observed in relation to near miss events in a number of studies. In one study, P300 and P3b were shown to be associated with false spatial feedback, while FRN activity was associated with unexpected negative feedback alone (Balconi and Crivelli 2010). This is consistent other studies that show that FRN activity related to fairness precedes the P300 signal, which has been associated with a state of uncertainty or dissonance (Yu et al. 2015). Together, this suggests that near miss events may quickly be identified by the player’s brain as a negative event, but the false or uncertain nature of the near miss may take longer to be processed or revealed. How the timing and magnitude of these neural events relates to the selection and initiation of the next gambling act (be it continued play, pausing for a distinct period of time, or ceasing of a play session) is something that will require further investigation. One potential avenue for this future work would be to explore the clarity of the near miss event as aversive, as it is expected that a stronger differentiation between FRN and P300 signals would be possible when the outcome of the game was more directly manipulated.

With regard to LDWs, a strong and clear picture of their effect on the gambler was found, despite being a topic of less concerted study. LDWs were consistently found to inflate win estimates and be a component of players’ preferred games. There was also some evidence, in the form of SCL response and self-report, to indicate that the number of credits awarded by the LDW is what induces the reinforcement of play, despite ongoing losses. Three studies also specifically identified that game sounds were important in this reinforcement process. However, only one study was explicitly assessed how LDWs are perceived by the player or are effecting the player, making it difficult to determine if the arousal present in the SCL and HRV responses are the product of excitement or are instead the product of a change in the level of effort or vigilance expended while gambling, as was introduced previously. Through further work differentiating the various modes of cognitive processing from the excitement of an LDW event, much stronger models could be established to describe and assess the effect of LDWs on healthy and problem gamblers alike, and could highlight new methods for producing a safe and healthy gambling environment. Studies on the neural substrates of the response to LDWs could also place the behavioural findings in a more general context, allowing a more direct comparison between the cognitive processing underlying both types of simultaneously reinforcing loss events—the near miss and the LDW—to be accounted for.

In both the study of near misses and LDWs, whether the effects differ in problem or at-risk populations remains to be seen. Only a few studies explored research questions related to non-healthy players. Of the studies that were identified, the research approach and question of interest varied widely, making it difficult to draw any strong conclusions on whether near misses or LDWs affect problem gamblers in any consistent way. However, some evidence was found to indicate that problem gamblers may have a reduced or suppressed emotional response to near misses. This was indicated by diffuse patterns of elevated activity in prefrontal cortex, amygdala, and striatum across a number of studies (Goldin et al. 2008). Many of the brain areas, such as the midbrain and striatum, have also been implicated in altered dopamine transmission and reception throughout the brain, something that appears relevant in gamblers as a whole (Bergh et al. 1997; Meyer et al. 2004). In two of the sampled studies, one on near misses and one on LDWs, some evidence was reported for players to be hyposensitive to wins and losses, neurologically (Lole et al. 2015) or in terms of SCL level (Lole et al. 2014). Problem gamblers were also found to prefer multiline slot machines more than non-problem gamblers. Taken as a whole, these works suggest that the complex emotional or reinforcing responses that problem gamblers have to near misses and LDWs may be blunted, potentially encouraging continued play when it is otherwise inadvisable. One reason for this may be that near misses and LDWs produce brief levels of excitement, causing spikes in the otherwise reduced response, sufficient to produce engagement and continued play, but insufficient to cause a change in behaviour, though this possibility requires further study to confirm.

The present review was limited by the inclusion of studies using a diverse number of techniques and approaches to studying the effects of near misses and LDWs. Given the number of studies identified investigating the topic of LDWs, this choice represented a practical necessity. However, within the topic of near misses, it remains possible that a more stringent review could establish with greater certainty the particular effect of near misses on the player through a statistically rigorous meta-analysis. As no review of this scope exists in the field of gambling studies at this time, we instead chose to describe and relate the effects of near misses and LDWs on the player to provide a current account of the effect of each mechanism and the current state of knowledge, however, rather than precisely target one specific research question. The current review was also limited by its inclusion of only peer-reviewed work. It remains possible that books, dissertations, or grey literature could provide more detail on the understanding of the effects of near misses and LDWs, given the multi-disciplinary nature of the field. However, these forms of media were excluded to ensure a consistent level of quality throughout the review.

In spite of these limitations, the current review provides an important foundation for future work in this area. The present systematic review on near misses and LDWs establishes how each of these two systematic characteristics of slot machines that appear to mislead the player into gambling through a variety of different mechanisms or outcome. Where clear evidence was found for near misses to be perceived as “almost winning”, reinforcing continued play through their surprising or exciting nature, LDWs appear to be viewed as a type of win more directly (despite actually being a loss). This systematic review provides a comprehensive description of the effects of near misses and LDWs, highlighting both consistent and inconsistent findings. Clear directions for future research were also provided, addressing topics of theoretical or conceptual importance to the understanding of EGM play and gambling behaviour as a whole and providing a framework for future work to build upon.

## Electronic supplementary material

Below is the link to the electronic supplementary material.

Supplementary material 1 (DOCX 21 kb)
