# Supplementary material for: The Effect of Losses Disguised as Wins and Near Misses in Electronic Gaming Machines: A Systematic Review
Source: J Gambl Stud. 2017 Apr 18;33(4):1241–60. doi: 10.1007/s10899-017-9688-0 (PMC5663799; doi:10.1007/s10899-017-9688-0)
Supplement: Supplementary file 1 — Supplementary material 1 (DOCX 21 kb) [file 10899_2017_9688_MOESM1_ESM.docx]

## Search Strategies

**Scopus Search**

- Performed November 20, 2015 by Sarah Brown, Liaison Librarian, University of Waterloo
- Retrieved 225 results

(TITLE-ABS-KEY(gambl* OR lottery OR gaming OR pokies OR poker OR slot OR slots OR "fruit machine" OR "fruit machines" OR casino OR casinos OR lotteries)) AND ((TITLE-ABS-KEY(near w/2 (miss OR misses OR win OR wins)) OR TITLE-ABS-KEY(loss* w/4 (win OR wins)) OR TITLE-ABS-KEY(multiline OR multi?line OR "multiple payline*") OR TITLE-ABS-KEY("just missed" OR "almost winning" OR "narrow win" OR "narrow wins" OR "just missing" OR "small win" OR "small wins" OR "fake win" OR "fake wins" OR "false win" OR "false wins") OR TITLE-ABS-KEY("weighted reel*" OR "unbalanced reel*" OR "virtual reel*")))

**PubMed**

- Performed November 20, 2015 by Jackie Stapleton, Liaison Librarian, University of Waterloo
- Retrieved 170 results

| #1 | (just[all fields] OR almost[all fields] OR fake[all fields] OR false[all fields] OR near[all fields] OR narrow[all fields] OR small[all fields]) AND (win[all fields] OR wins[all fields] OR winning[all fields]) |
| --- | --- |
| #2 | “weighted reel*”[all fields] OR “unbalanced reel*”[all fields] OR "near miss"[All Fields] OR "near misses"[All Fields] OR “virtual reel*”[all fields] OR (losses[tiab] AND wins[tiab]) OR multiline[All Fields] OR "multi line"[All Fields] OR “multiple paylines”[all fields] |
| #3 | #1 OR #2 |
| #4 | gambl*[all fields] or gambling[mesh] OR lottery[all fields] OR gaming[all fields] OR pokies[all fields] OR poker[all fields] OR slot[all fields] OR slots[all fields] OR "fruit machine*"[all fields] OR casino[all fields] OR casinos[all fields] OR lotteries[all fields] |
| #5 | #3 AND #4 |

**PsycInfo Search**

- Performed November 20, 2015 by Sarah Brown, Liaison Librarian, University of Waterloo
- Retrieved 221 results

( ( AnyField:( " loss" NEAR/4 ( " win" OR " wins" ) ) OR AnyField:( " losses" NEAR/4 ( " win" OR " wins" ) ) OR AnyField:( multiline OR multi-line OR " multi line" OR " multiple paylines" OR " multiple payline" ) ) OR ( AnyField:( " weighted reel" OR " weighted reels" OR " unbalanced reel" OR " unbalanced reels" OR " virtual reel" OR " virtual reels" ) ) OR ( AnyField:( " just missed" OR " almost winning" OR " just missing" OR " narrow win" OR " narrow wins" ) OR AnyField:( " fake win" OR " fake wins" OR " false win" OR " false wins" ) OR AnyField:( " small win" OR " small wins" ) ) OR ( AnyField:( " near" NEAR/2 ( " miss" OR " misses" OR " win" OR " wins" ) ) ) ) AND ( AnyField:( gambl* OR lottery OR gaming OR pokies OR poker OR slots OR slot OR " fruit machine" OR " fruit machines" OR casino OR casinos OR lotteries ) )

**ProQuest Sociology Collection**

- Performed November 20, 2015 by Sarah Brown, Liaison Librarian, University of Waterloo
- ProQuest Sociology collection included: ERIC, International Bibliography of the Social Sciences, PILOTS (Published International Research on Traumatic Stress), ProQuest Sociology, Social Services Abstracts, Sociological Abstracts
- Retrieved 132 results

(gambl* OR lottery OR gaming OR pokies OR poker OR slot OR slots OR "fruit machine" OR "fruit machines" OR casino OR casinos OR lotteries) AND (("weighted reel" OR "weighted reels" OR "unbalanced reel" OR "unbalanced reels" OR "virtual reel" OR "virtual reels") OR ("near" NEAR/2 ("miss" OR "misses" OR "win" OR "wins")) OR (("just missed" OR "just missing" OR "almost winning" OR "narrow win" OR "narrow wins" OR "fake win" OR "fake wins") OR ("false win" OR "false wins" OR "small wins" OR "small win" )) OR (loss* NEAR/4 (win OR wins)) OR (multiline OR multi?line OR "multiple payline" OR "multiple paylines"))

All keywords searched in anywhere fields

**GREO Knowledge Respository**

- Performed November 23, 2015 by Jackie Stapleton, Liaison Librarian, University of Waterloo
- Database: [www.greo.ca](http://www.greo.ca) (synopses collection only)
- Retrieved 54 unique records

| Search strategy | # results |
| --- | --- |
| Near miss (will find near misses) | 26 |
| Weighted reel | 0 |
| Unbalanced reel | 0 |
| False win | 14 |
| Fake win | 1 |
| Loss disguise | 10 |
| LDWs | 6 |
| multiline | 12 |
| total | 64  (54 unique records) |
